# Supplementary figures and images for: The efficacy and safety of topical wound oxygen therapy for chronic refractory wounds at high altitude: Protocol for a randomized controlled clinical trial
Source: PLoS One. 2025 Jul 10;20(7):e0324475. doi: 10.1371/journal.pone.0324475 (PMC12244751; doi:10.1371/journal.pone.0324475)

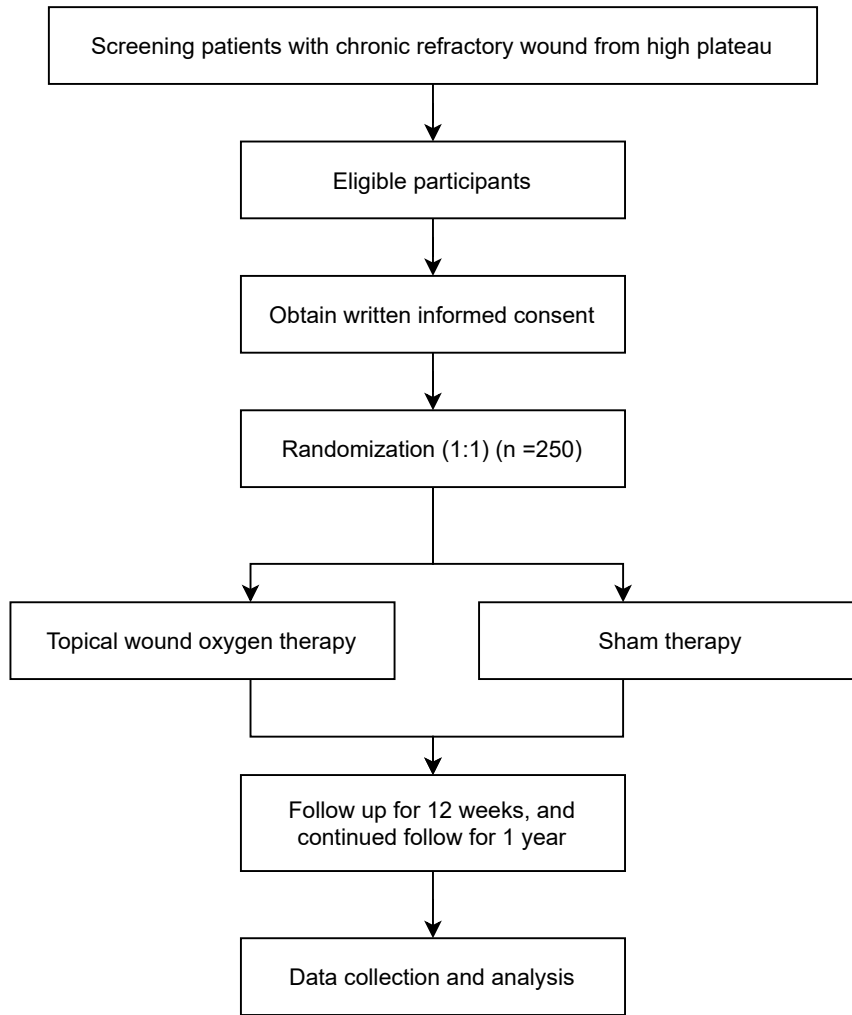

Supplement: S1 File — (PDF) [file pone.0324475.s001.pdf]
