# Supplementary material for: The efficacy and safety of topical wound oxygen therapy for chronic refractory wounds at high altitude: Protocol for a randomized controlled clinical trial
Source: PLoS One. 2025 Jul 10;20(7):e0324475. doi: 10.1371/journal.pone.0324475 (PMC12244751; doi:10.1371/journal.pone.0324475)
Supplement: S3 File — (PDF) [file pone.0324475.s003.pdf]

内 部 资 料
注 意 保 密

生物医学伦理研究方案
（干预性临床研究）

局部创面氧疗法治疗高原慢性难愈性创面者的有效性与安全性临床研究方案

研究单位：四川大学华西医院

项目负责人（签名）：冉兴无

承担科室：内分泌代谢科

联系电话：189980601305

组长单位：四川大学华西医院

参加单位：四川大学华西医院

研究年限：2024 年 10 月 —— 2026 年 07 月

版本号：V3.0

版本日期：2023 年 11 月 25 日

方 案 摘 要

研究设计<br>(可多选)	☐病例对照研究 ☐队列研究 ☐横断面研究<br>☑ 随机对照研究 ☐应用盲法 ☐其他：
研究类型<br>（请根据项目类型勾选）	（A 类：高风险）<br>☐III类临床新技术（安全性、有效性确切，技术难度大、风险高）<br>☐ 特殊人群研究（儿童、孕妇、智力低下者、精神障碍受试者等）<br>☐ 超药物说明书研究（☐超适应症 ☐超给药途径 ☐超剂量 ☐超年龄 ☐超禁忌症 ☐超人群 ☐其他，请说明： ）<br>☐ 超器械说明书研究（☐超适应症 ☐使用范围 ☐超禁忌症 ☐超人群 ☐其他，请说明： ）<br>☐ 其他（研究者判定，请说明： ）<br><br>（B 类：中风险）<br>☐ 上市后生物制剂研究（预防用和治疗用）<br>☐ 上市后治疗性疫苗研究<br>☐ 上市后罕见病药物研究<br>☐ II类临床新技术（安全性、有效性确切，有一定技术难度，有一定医疗风险和伦理风险）<br>☐ 其他（研究者判定，请说明： ）<br><br>（C 类：低风险）<br>☐ 已上市 5 年药物研究（包括化药、仿制药等）<br>☑ 已上市器械研究（含 AI，影像软件）<br>☐ I 类临床新技术（安全性、有效性确切，技术难度低、几乎不存在伦理风险的医疗技术）<br>☐ 其他（研究者判定，请说明： ）
病例总数	250例
风险/受益分析	
风险判断	☐不大于最小风险 ☑ 大于最小风险<br>最小风险：指试验中预期风险的可能性和程度不大于日常生活、或进行常规体格检查或心理测试的风险

一、研究背景

慢性创面是指由各种原因引起的经过 1 个月以上标准化治疗仍未痊愈，或者没有愈合趋势的创面，包括糖尿病创面、感染性创面、压力性创面、创伤性创面等 1。慢性创面因病因复杂、治疗周期长、花费高，且易复发、易致残等原因，一直是当今医疗领域较为棘手的难题 2。高原慢性难愈性创面一直是高原医学关注的重点。

高原地区由于低压、低氧环境，创面持续的低氧低灌注与炎症反应，以及相对较差的卫生条件，导致高原地区的慢性创面与平原地区具有独特的病理生理特点 3。祁万乐等研究者在青海省人民医院的回顾性研究中发现高原地区老年慢性创面的主要病因是糖尿病足，占比 51.9%，其次为压疮、手术伤口感染、创伤性溃疡、静脉性溃疡和动脉性溃疡 4。高原地区的创面细菌感染特点和耐药性也不同于平原地区 5。在高原地区，创面更易出现脂肪液化、切口感染、伤口血肿以及迁延不愈等问题 6。此外，高原慢性心肺疾病等基础疾病会促进难愈合创面的发展。

氧是伤口愈合所必需的成分，在创面愈合各阶段均起着重要作用。炎症期，活性氧（ROS）的生成促进吞噬细胞杀伤病原体，抑制微生物生长，清除坏死组织；增值期，氧对于胶原蛋白合成、细胞外基质沉积，血管形成具有重要作用。局部组织缺氧是限制伤口愈合的一个重要因素 7。我们前期的研究也证实，缺氧可导致缺氧诱导因子（HIF-2α）的失调，导致创面愈合延迟 8。炎症、水肿、疼痛等导致血管收缩，糖尿病周围动脉病变导致肢体血供受损，创面感染导致耗氧增加，均容易导致慢性创面组织缺氧。高原地区，低压低氧环境可能进一步加重慢性创面组织缺氧。

20 世纪 60 年代开始在临床上将氧气用于促进伤口愈合。高压氧治疗是将患者置于高压环境中，吸入纯氧的治疗方法。一项纳入 12 项随机对照研究（577 例患者）的系统评价发现：高压氧可显著改善糖尿病足溃疡创面愈合与降低截肢风险，减少下肢静脉性溃疡的面积 9。但高压氧设备在全球范围内广泛应用和发展受到了诸多限制，如设备的可用性、禁忌症、需要转移患者的不便性等。

为了解决这些不足之处，引入了局部氧气治疗。临床前动物研究显示，局部氧气治疗，可增加经皮氧分压、增加血管内皮生长因子、改善血管形成、组织重塑，促进创面愈合 10-12。临床对照研究显示，局部创面氧气治疗明显改善 12 周

时溃疡愈合率（76% vs. 46%，P＜0.001)13，缩短创面愈合时间（56 天 vs. 93 天）14。一项全球多中心随机双盲对照试验结果显示，局部创面氧气治疗明显改善创面愈合率（OR 6， 97.8%CI 1.44-24.93，P =0.004）15。然而，目前尚没有研究探讨局部创面氧疗在治疗久居高原地区患者慢性难愈性创面中的作用。

本研究项目拟通过随机对照研究评估局部创面氧疗在来自高原地区患者的慢性难愈性创面治疗的有效性与安全性，为高原慢性难愈合性创面的治疗提供全新有效安全的治疗方案。

二、研究目的

1. 主要目的：通过随机对照研究明确局部创面氧疗在来自高原地区患者慢性难愈性创面的作用，为高原慢性难愈性创面的治疗提供一种全新有效安全的治疗方案。

三、研究设计、方法与研究步骤

1. 研究设计

本研究拟通过设计随机对照临床试验，评估局部创面氧疗对来自高原地区患者慢性难愈性创面治疗的有效性与安全性。本单位负责研究设计与实施，项目合作单位负责协助部分病例收集与随访。

2. 研究方法

经单位伦理委员会批准，患者签署知情同意后，纳入符合纳排标准的来自高原地区的慢性难愈性创面患者，通过在标准创面治疗基础上给予局部创面氧疗或对照治疗，评估局部创面氧疗在久居高原患者慢性难愈性创面治疗的有效性与安全性。

（1） 样本量计算

Blackman（Ostomy Wound Manage. 2010;56(6):24-31.）的研究显示糖尿病足标准治疗组的 12 周溃疡愈合率 42.8%，局部创面氧疗组创面愈合率 85.2%。我们前期研究中，糖尿病标准治疗组的创面愈合率 69%（Wound Repair Regen. 2015;23(4):495-505.）。按照标准治疗组 12 周的创面愈合率 60%，使用局部创面氧疗预计提高 30%的创面愈合率，检验水准（α）为 0.05，检验效能（power）

为 0.8，在 PASS 15 软件中采用随机对照试验（两组率）比较的方法计算样本量，得知实验组与对照组各需 100 例患者；按照 20%的失访率计算，共需纳入 250 例患者。

（2） 纳入标准：① 年龄在 18-80 岁之间；② 来自高原地区（居住地海拔 2500m 以上）；③ 慢性难愈性创面（创面 4 周未愈合）：糖尿病慢性下肢皮肤溃疡，下肢静脉功能不全性溃疡；④ 恰当的血供，ABI≥0.6，经皮氧分压＞30mmHg；⑤ 溃疡面积 1-20cm2。

（3） 排除标准：① 肢体坏疽；② 骨髓炎；③ 恶性肿瘤；④ HIV 阳性；⑤严重的心脏、肝脏、肾脏、呼吸系统、神经系统等疾病；⑥ 长期使用类固醇或其它免疫抑制剂；⑦ 妊娠或者治疗前后 3 个月有妊娠计划，以及哺乳期的女性；⑧ 有精神疾病或严重认知功能障碍，酒精或药物滥用，不能配合治疗者。

（4） 临床资料收集

收集入选患者的性别、年龄、受教育程度、家族史、个人史等人口学资料；糖尿病、高血压、冠心病、脑梗塞、脂代谢紊乱、肿瘤等合并疾病，以及疾病病程，发病经过，抗生素使用历史，足部 X 片，血管彩超，踝肱指数等。

（5） 随机化分配

符合纳入排除标准的患者，按 1：1 的比例随机分配入标准治疗联合局部创面氧疗组（治疗组）或标准治疗联合假性给氧组（对照组）。

（6） 干预措施

慢性创面的标准治疗：包括血糖、血压、血脂控制，抗血小板聚集、抗凝、调脂、抗感染等内科治疗；运动锻炼；减压、清创；以及富血小板凝胶，负压吸引术等治疗。

治疗组：在慢性创面标准治疗方案基础上，将在病房床旁使用爱尔兰欧弟氧疗公司的下肢伤口氧疗仪（G00001，设备的器械注册证见附件）进行局部创面氧疗（0～50mbar 的循环加压氧；氧气由 10L/min 的制氧机提供；持续时间 90 分钟；每天 1 次，每周 5 天）。

对照组：在慢性创面标准治疗方案基础上，加用局部创面氧疗装置，但是使用空气作为气体来源，不接通氧气源。

（7） 治疗与随访

本研究治疗时间 12 周，随访时间 1 年。所有患者随机分配入治疗组或对照组后，给予相应的治疗，直至创面愈合或满 12 周。于每次清创、换药时，采用数码相机拍照（至少 1 周 1 次），进行足溃疡面积测定；定期进行 ABI 测定，疼痛评估（Visual-Analog Scale 量表）。于开始治疗的第 4、8、12、24、52 周进行随访，评估创面愈合情况，溃疡复发与截肢等。

（8） 结局指标

主要结局指标：12 周创面愈合率

次要结局指标：12 周溃疡面积减少率，溃疡愈合时间，溃疡复发率，截肢率，疼痛评估以及其它不良反应。

（9） 统计分析

采用 Stata 13 进行统计分析。主要结局指标，采用卡方检验评估 12 周时创面愈合率。应用 Logistc 回归分析影响创面愈合的可能混杂因素。次要结局指标，采用独立样本 t 检验评估 12 周溃疡面积减少率与溃疡愈合时间，采用卡方检验评估溃疡复发率与截肢率。同时采用 Kaplan-Meire 生存分析比较两组患者创面愈合。$P<0.05$ 为差异具有统计学意义。

3. 研究步骤

本研究的技术路线如图 1 所示。

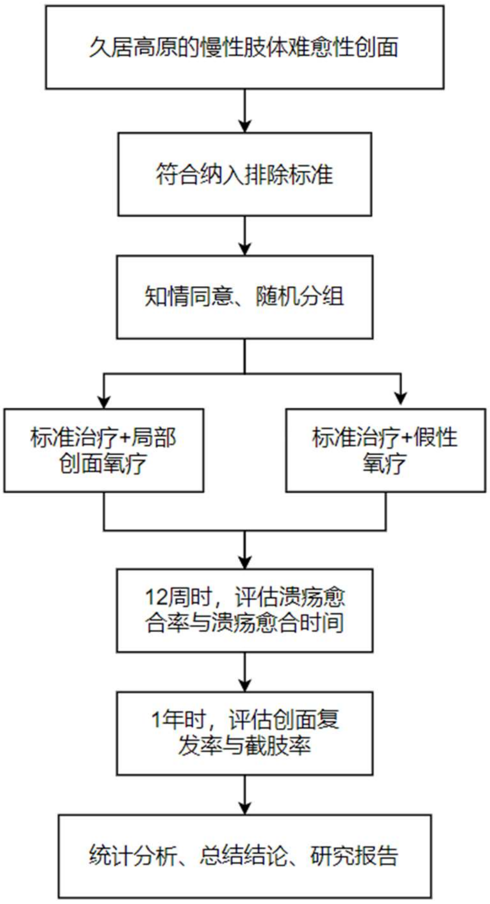

四、病例选择

1. 入选标准

① 年龄在 18-80 岁之间；② 来自高原地区（居住地海拔 2500m 以上）；③ 慢性难愈性创面（创面 4 周未愈合）：糖尿病慢性下肢皮肤溃疡，下肢静脉功能不全性溃疡；④ 恰当的血供，ABI≥0.6，经皮氧分压＞30mmHg；⑤ 溃疡面积 1-20cm2。

2. 排除标准

① 肢体坏疽；② 骨髓炎；③ 恶性肿瘤；④ HIV 阳性；⑤严重的心脏、肝脏、肾脏、呼吸系统、神经系统等疾病；⑥ 长期使用类固醇或其它免疫抑制剂；⑦ 妊娠或者治疗前后 3 个月有妊娠计划，以及哺乳期的女性；⑧ 有精神疾病或严重认知功能障碍，酒精或药物滥用，不能配合治疗者。

3. 终止研究标准

① 患者自愿退出试验；② 出现严重不良反应导致治疗中断者；③ 试验开始经过 4 周治疗溃疡无反应者（溃疡面积缩小<20%）；④ 创面表现出严重临床感染的迹象，需要立即手术干预；⑤ 目标病灶所在患肢出现大截趾/肢指征者。

五、可供选择的其他诊疗方法

近年来，多种慢性创面的局部治疗方法，如负压吸引闭式引流、重组人表皮生长因子、鼠神经成纤维细胞生长因子、自体富血小板凝胶等逐渐在临床中得到应用，但治疗效果仍有限且治疗费用较高。

六、检测项目与检测时点

本研究主要通过定期（1，2，4，8，12 周）测量创面面积，评估疗效。

七、疗效评定标准

1.有效性评估：根据创面愈合率和愈合时间进行。在治疗前和治疗后每周测量创面面积并计算创面愈合率。使用无菌标准透明方格薄膜勾边，直接计算创面

面积或对薄膜进行数码照相，用 Image J 医学图像分析软件电脑自动计算数码照相的创面面积。

（1）创面愈合率=（治疗初始面积-治疗后面积）/治疗初始面积*100%。愈合效果：①痊愈　创面愈合率≥90%；②显效　60% ≤创面愈合率<90%；③进步　30% ≤创面愈合率<60%；④无效　创面愈合率＜30%

（2）愈合时间：创面被上皮完全覆盖的时间（天）

2.安全性评价：

（1）记录所有不良反应。

（2）不良反应发生率=出现某不良反应的患者数量/该组患者总数*100%

八、不良事件的观察、记录和处置

局部伤口氧疗自 20 世纪 60 年代以来被用于治疗急性及慢性伤口。下肢伤口氧疗仪的设计和制造的技术已经成熟。由于这是一种无创疗法，提供的压力略高于环境压力(0-50mbar) ，因此是一种患者无痛苦、无创伤、安全的治疗方法。

1.治疗前：对下肢伤口氧疗仪氧疗舱、脚套或者一次性使用肢体氧疗袋进行清洁消毒，以减少任何潜在的交叉感染风险。建议在治疗前去除附在伤口上的绷带、敷料或者药膏。在使用设备进行治疗之前，应根据标准伤口护理方法对伤口进行清洁或清创。

2.治疗中：根据医生处方设置治疗时间和治疗压力：（1）如果治疗压力超过限制范围，设备会发出高压报警，阀门会立即关闭，舱室压力排放到大气中自动泄压。（2）如果舱室中的治疗压力在 90 秒内未达到，表明设备有漏气，会发出低压警报，需要检查所有管道的连接是否正确；检查脚套密封性及舱门的密封性，如有必要进行更换；适当调整患者体位。（3）治疗时确保房间通风，请勿吸烟、艾灸或使用明火。（4）治疗期间，对患者进行伤口评估、清创、收集伤口照片、做好治疗情况记录。

3.治疗后：患者应遵循临床医生的建议，并按照标准护理程序使用合适的敷料，按时换药，做好治疗后的跟进反馈。对一次性脚套或一次性肢体氧疗袋等进行妥善处理。消毒舱室内外及调节器的外表面，包括气氧气软管和电源线，遵循生产厂家要求进行清洁消毒。

如果发生不良反应或严重不良反应，将根据法律规定进行不良反应的报告与处理。

九、研究的质量控制与质量保证

研究团队将严格进行实验室指标的检测、严格执行 SOP 的执行要求和 GCP 的规定、对研究者进行严格的培训、努力提高受试者依从性、准确、真实、完整、及时地收集数据，合理进行数据的整理分析、实时监查研究过程，完成总结报告。

十、数据安全监查

临床研究将根据风险大小制定相应的数据安全监察计划。所有不良事件均详细记录，恰当处理并追踪直到妥善解决或病情稳定，按照规定及时向伦理审查委员会、主管部门、申办者和药品监督管理部门报告严重不良事件与非预期事件等；主要研究者定期对所有不良事件进行累积性回顾，必要时召开研究者会议评估研究的风险与受益；双盲试验必要时可以进行紧急揭盲，以确保受试者安全与权益。

十一、统计学处理

采用 Stata 13 进行统计分析。主要结局指标，采用卡方检验评估 12 周时创面愈合率。应用 Logistc 回归分析影响创面愈合的可能混杂因素。次要结局指标，采用独立样本 t 检验评估 12 周溃疡面积减少率与溃疡愈合时间，采用卡方检验评估溃疡复发率与截肢率。同时采用 Kaplan-Meire 生存分析比较两组患者创面愈合。$P < 0.05$ 为差异具有统计学意义。

十二、临床研究伦理原则与要求

临床研究将遵循世界医学大会《赫尔辛基宣言》和中华人民共和国国家卫生和计划生育委员会《涉及人的生物医学研究伦理审查办法》等相关规定，具体落实知情同意，保护隐私，研究免费与补偿，控制风险，特殊受试者保护和研究相关损害的赔偿原则与要求。在研究开始之前，由伦理审查委员会批准该试验方案

后才实施临床研究。每一位受试者入选本研究前，研究者有责任向受试者或/和其法定代理人完整、全面地介绍本研究的目的、程序和可能的风险，并签署书面知情同意书，应让受试者知道他们参加临床研究完全是自愿的，他们可以拒绝参加或在试验的任何阶段随时退出本研究而不会受到歧视和报复，其医疗待遇与权益不受影响。知情同意书应作为临床研究文件保留备查，切实保护受试者的个人隐私与数据机密性。

十三、研究进度

2024 年 10 月-2025 年 10 月：筛选招募符合研究条件的久居高原的慢性难愈性创面患者，签署知情同意后纳入研究。根据研究计划，全面收集受试者的基线资料，包括人口学数据、合并疾病与并发症、生化指标等检查。根据入组后方案，针对患者进行相应的干预治疗，评估创面愈合率、愈合时间等结局指标。

2025 年 10 月-2026 年 4 月：继续招募符合条件的受试者，完善研究。对已纳入研究的患者，根据预设的随访时间进行随访评估干预治疗后的创面复发率、截肢率等。

2026 年 04 月-2026 年 07 月：继续完善病例的随访。分析实验结果，数据分析，撰写总结材料及论文，进行课题结题、成果鉴定。

十四、参加人员

姓名	职称	专业	任务	GCP 培训证书
冉兴无	主任医师	内分泌	方案设计与技术指导	有
陈大伟	副主任医师	内分泌	筛选与治疗	有
高赟	副主任医师	内分泌	筛选与治疗	有
陈利鸿	主治医师	内分泌	筛选和治疗	有
李艳	助理研究员	内分泌	筛选和治疗	有
吴静	无	内分泌	筛选和随访	有
方怡轩	无	内分泌	筛选和随访	有

十五、主要参考文献

1 廖新成, 郭光华. 慢性难愈性创面的分类鉴别及临床评估. *中华损伤与修复杂志：电子版* 2017:303-5.

2 Cheng B, Jiang Y, Fu X, Hao D, Liu H, Liu Y, et al. Epidemiological characteristics and clinical analyses of chronic cutaneous wounds of inpatients in China: Prevention and control. *Wound Repair Regen* 2020;**28**:623-30.

3 何斯, 严辰媛, 刚乔健, 贾彩霞, 医学综述 哈 J. 高原地区难愈合创面病理变化和特点. 2022;**28**:2746-50.

4 祁万乐, 卓么加, 田琰, 达娃卓玛, 马子英, 安亚南, et al. 高原地区老年慢性难愈合创面患者流行病学调查分析. *中华损伤与修复杂志：电子版* 2021;**16**:6.

5 于小惠, 丁巳娟, 何冲, 冯东方, 肖静, 陈红, et al. 高原低氧环境下烧伤科患者创面普通培养细菌分布特点和耐药性分析. 2022;**43**:2841-45.

6 廉国锋, 陈郁, 陈兴书, 人民军医 罗 J. 部队高原疾病及灾害防治系列研究(7) 高原地区伤口愈合不良医学地理特征及防治措施. 2019:93-6.

7 Gottrup F, Dissemond J, Baines C, Frykberg R, Jensen P, Kot J, et al. Use of Oxygen Therapies in Wound Healing. *J Wound Care* 2017;**26**:S1-s43.

8 Chen L, Gao Y, Li Y, Wang C, Chen D, Gao Y, et al. Severe Intermittent Hypoxia Modulates the Macrophage Phenotype and Impairs Wound Healing Through Downregulation of HIF-2α. *Nature and science of sleep* 2022;**14**:1511-20.

9 Kranke P, Bennett MH, Martyn-St James M, Schnabel A, Debus SE, Weibel S. Hyperbaric oxygen therapy for chronic wounds. *Cochrane Database Syst Rev* 2015;**2015**:Cd004123.

10 Gordillo GM, Roy S, Khanna S, Schlanger R, Khandelwal S, Phillips G, et al. Topical oxygen therapy induces vascular endothelial growth factor expression and improves closure of clinically presented chronic wounds. *Clin Exp Pharmacol Physiol* 2008;**35**:957-64.

11 Fries RB, Wallace WA, Roy S, Kuppusamy P, Bergdall V, Gordillo GM, et al. Dermal excisional wound healing in pigs following treatment with topically applied pure oxygen. *Mutat Res* 2005;**579**:172-81.

12 Asmis R, Qiao M, Zhao Q. Low flow oxygenation of full-excisional skin wounds on diabetic mice improves wound healing by accelerating wound closure and reepithelialization. *Int Wound J* 2010;**7**:349-57.

13 Tawfick W, Sultan S. Does topical wound oxygen (TWO2) offer an improved outcome over conventional compression dressings (CCD) in the management of refractory venous ulcers (RVU)? A parallel observational comparative study. *Eur J Vasc Endovasc Surg* 2009;**38**:125-32.

14 Blackman E, Moore C, Hyatt J, Railton R, Frye C. Topical wound oxygen therapy in the treatment of severe diabetic foot ulcers: a prospective controlled study. *Ostomy Wound Manage* 2010;**56**:24-31.

15 Frykberg RG, Franks PJ, Edmonds M, Brantley JN, Téot L, Wild T, et al. A Multinational, Multicenter, Randomized, Double-Blinded, Placebo-Controlled Trial to Evaluate the Efficacy of Cyclical Topical Wound Oxygen (TWO2) Therapy in the Treatment of Chronic Diabetic Foot Ulcers: The TWO2 Study. *Diabetes Care* 2020;**43**:616-24.
