# Supplementary material for: The efficacy and safety of topical wound oxygen therapy for chronic refractory wounds at high altitude: Protocol for a randomized controlled clinical trial
Source: PLoS One. 2025 Jul 10;20(7):e0324475. doi: 10.1371/journal.pone.0324475 (PMC12244751; doi:10.1371/journal.pone.0324475)
Supplement: S4 File — (PDF) [file pone.0324475.s004.pdf]

Internal Information

Biomedical Ethics Research Program (Interventional Clinical Study)

Clinical study protocol on the effectiveness and safety of local wound oxygen therapy in the treatment of patients with chronic refractory wounds at high altitude

Research Unit: West China Hospital, Sichuan University

Project Leader (Signed): Ran Xingwu

Department: Department of Endocrinology and Metabolism

Contact number: 189980601305

Team Leader Unit: West China Hospital, Sichuan University

Participating unit: West China Hospital, Sichuan University

Research period: October 2024 - July2026

Version: V3.0

Version date: November 25, 2023

Executive Summary

Study design (Multiple selections allowed)	☐ case-control studies ☐ cohort studies ☐ cross-sectional studies<br>☑ Randomized controlled studies ☐ application blinding ☐ other:
Type of study (Please select according to the project type)	(Category A: High Risk)<br>☐ Class III new clinical technology (safety and efficacy, technical difficulty and high risk)<br>☐ Special population studies (children, pregnant women, people with mental retardation, subjects with mental disorders, etc.)<br>☐ Off-label study (☐ off-label ☐ overdose ☐ overdose ☐ overage ☐ ultra-contraindications ☐ supercrowd ☐ Others, please specify:)<br>☐ Ultra-device instruction manual study (☐ off-label ☐ scope of use ☐ ultra-contraindications ☐ super-population ☐Other, please specify:)<br>☐ Other (as judged by the investigator, please specify:)<br><br>(Category B: Medium Risk)<br>☐ Post-marketing biologics studies (prophylactic and therapeutic)<br>☐ Post-marketing therapeutic vaccine studies<br>☐ Post-marketing rare disease drug studies<br>☐ Class II clinical new technology (safety, efficacy, technical difficulty, medical risk and ethical risk).<br>☐ Other (as judged by the investigator, please specify:)<br><br>(Category C: Low Risk)<br>☐ Pharmaceutical research that has been on the market for 5 years (including chemical drugs, generic drugs, etc.)<br>☑ Commercialized device research (including AI, imaging software)<br>☐ Class I new clinical technology (medical technology with definite safety, efficacy, low technical difficulty, and almost no ethical risk).<br>☐ Other (as judged by the investigator, please specify:)
Total number	250 cases

of cases	
Risk/benefit analysis	
Risk Judgment	☐ is not greater than the minimum risk ☑ is greater than the minimum risk<br>Minimal risk: Refers to the likelihood and degree of risk expected in the test that is no greater than the risk of daily living, or routine physical examination or psychological testing

1. Background

Chronic wounds are caused by various causes that have not healed after more than 1 month of standardized treatment.or wounds that have no tendency to heal, including diabetic wounds, infectious wounds, pressure wounds, traumatic wounds, etc1。Chronic wounds have always been a difficult problem in the medical field today due to complex etiology, long treatment cycle, high cost, easy recurrence and disability2。Chronic refractory wounds at high altitude have always been the focus of high-altitude medicine.

Due to the low pressure and low oxygen environment, the continuous hypoxia, hypoperfusion and inflammatory response of the wound, and the relatively poor sanitary conditions, the chronic wounds in the plateau area and the plain area have unique pathophysiological characteristics3。 In a retrospective study of Qinghai Provincial People's Hospital, Qi Wanle et al. found that diabetic foot was the main cause of chronic wounds in the elderly in the plateau area, accounting for 51.9%, followed by pressure ulcers, surgical wound infections, traumatic ulcers, venous ulcers and arterial ulcers4。 The characteristics of bacterial infection and drug resistance in the plateau area are also different from those in the plain area5。 In highland areas, wounds are more likely to suffer from liquefaction of fat, incision infection, wound hematoma, and prolonged healing6。In addition, underlying diseases such as chronic cardiopulmonary diseases at high altitude will promote the development of refractory wounds.

Oxygen is an essential ingredient for wound healing and plays an important role in all stages of wound healing. During the inflammatory phase, the production of reactive oxygen species (ROS) promotes phagocytic cells to kill pathogens, inhibit the growth of microorganisms, and remove necrotic tissues. During the proliferation phase, oxygen plays an important role in collagen synthesis, extracellular matrix deposition, and vascular formation. Local tissue hypoxia is an important factor limiting wound healing7。 Our previous studies have also confirmed that hypoxia can lead to dysregulation of hypoxia-inducible factor (HIF-2α), resulting in delayed wound healing8。Inflammation, edema, pain, etc. lead to vasoconstriction, diabetic peripheral arterial lesions lead to damage to the blood supply of limbs, and wound infection leads

to increased oxygen consumption, all of which can easily lead to chronic wound tissue hypoxia. In plateau areas, the hypobaric and hypoxic environment may further aggravate chronic wound tissue hypoxia.

In the 60s of the 20th century, oxygen was clinically used to promote wound healing. Hyperbaric oxygen therapy is a treatment in which a patient is placed in a high-pressure environment and inhaled pure oxygen. A systematic review of 12 randomized controlled studies (577 patients) found that hyperbaric oxygen significantly improved diabetic foot ulcer wound healing, reduced the risk of amputation, and reduced the size of venous leg ulcers9。 However, the widespread application and development of hyperbaric oxygen equipment around the world has been limited by many limitations, such as the availability of equipment, contraindications, and the inconvenience of needing to transfer patients.

To address these deficiencies, local oxygen therapy was introduced. Preclinical animal studies have shown that local oxygen therapy can increase transcutaneous oxygen partial pressure, increase vascular endothelial growth factor, improve vascularization, tissue remodeling, and promote wound healing10-12。 In a controlled clinical study, local wound oxygen therapy significantly improved ulcer healing at 12 weeks (76% vs. 46%, $P < 0.001$)13to reduce wound healing time (56 versus 93 days)14。 Results from a global multicenter randomized double-blind controlled trial showed that local wound oxygen therapy significantly improved wound healing (OR 6, 97.8%CI 1.44-24.93, $P =0.004$)15。 However, there are no studies to explore the role of local wound oxygen therapy in the treatment of chronic refractory wounds in patients living in highland areas for a long time.

This research project aims to evaluate the efficacy and safety of local wound oxygen therapy in the treatment of chronic refractory wounds in patients from plateau areas through randomized controlled studies, and to provide a new, effective and safe treatment option for the treatment of chronic refractory wounds at plateau.

2. Purpose of the study

Main objective: To clarify the role of local wound oxygen therapy in patients with

chronic refractory wounds from plateau areas through randomized controlled studies, and to provide a new, effective and safe treatment plan for the treatment of chronic refractory wounds at plateau.

3. Research design, methods and research procedures

3.1 Study design

This study intends to design a randomized controlled clinical trial to evaluate the efficacy and safety of local wound oxygen therapy for the treatment of chronic refractory wounds in patients from plateau areas. This unit is responsible for the design and implementation of the study, and the project partner is responsible for assisting in the collection and follow-up of some cases.

3.2 Research Methods:

With the approval of the ethics committee of the unit and the signing of informed consent, patients with chronic refractory wounds from plateau areas who meet the criteria for inclusion were included, and the efficacy and safety of local wound oxygen therapy in the treatment of chronic refractory wounds in patients living on plateau for a long time were evaluated by giving local wound oxygen therapy or control treatment on the basis of standard wound treatment.

（1） Sample size calculations

Blackman（Ostomy Wound Manage. 2010; 56(6):24-31.) showed that the 12-week ulcer healing rate was 42.8% in the standard treatment group of diabetic foot and 85.2% in the local wound oxygen therapy group. In our previous study, the wound healing rate in the standard diabetes treatment group was 69% (Wound Repair Regen. 2015; 23(4):495-505.）。According to the 12-week wound healing rate of 60% in the standard treatment group, the wound healing rate was expected to be increased by 30% with local wound oxygen therapy, the test level (α) was 0.05, and the test efficacy (power) was 0.8. Based on a 20% loss to follow-up rate, a total of 250 patients were included.

（2） Inclusion Criteria: (1) Age between 18-80 years; ② From the highland region(Altitude of residence.) 2500m or more）； (3) Chronic refractory wounds (wounds that have not healed for 4 weeks): diabetic chronic lower limb skin ulcers,

lower limb venous insufficiency ulcers; (4) Appropriate blood supply, ABI ≥ 0.6, transcutaneous partial pressure of oxygen > 30mmHg; (5) Ulcer area 1-20cm2.

（3） Exclusion Criteria: (1) limb gangrene; (2) osteomyelitis; (3) malignant tumors; (4) HIV-positive; (5) Serious heart, liver, kidney, respiratory system, nervous system and other diseases; (6) Long-term use of steroids or other immunosuppressants; (7) Pregnant or women who plan to become pregnant 3 months before or after treatment, and who are breastfeeding; (8) Those who have mental illness or severe cognitive impairment, alcohol or drug abuse, and cannot cooperate with treatment.

（4） Clinical data collection

Demographic data such as gender, age, education level, family history, and personal history of the selected patients were collected. Diabetes, hypertension, coronary heart disease, cerebral infarction, lipid metabolism disorders, tumors and other comorbid diseases, as well as the course of the disease, the onset of the disease, the history of antibiotic use, foot X-ray, vascular color ultrasound, ankle-brachial index, etc.

（5） Randomization allocation

Patients who met the inclusion criteria were randomly assigned to standard care plus local wound oxygen therapy (treatment group) or standard treatment combined with pseudo-oxygen administration (control group) in a 1:1 ratio.

（6） Interventions

Standard treatment of chronic wounds: including blood glucose, blood pressure, blood lipid control, antiplatelet aggregation, anticoagulation, lipid regulation, anti-infection and other medical treatments; athletic exercise; decompression, debridement; as well as platelet-rich gels, negative pressure aspiration and other treatments.

Treatment group: On the basis of the standard treatment plan for chronic wounds, local wound oxygen therapy (0 ~ 50mbar circulating pressurized oxygen; Oxygen is provided by a 10L/min oxygen concentrator; Duration: 90 minutes; 1 time per day, 5 days a week).

Control group: On the basis of the standard treatment plan for chronic wounds, a local wound oxygen therapy device was added, but air was used as a gas source and no

oxygen source was connected.

（7） Treatment and follow-up

The duration of treatment in this study was 12 weeks, and the follow-up time was 1 year. All patients were randomly assigned to the treatment group or the control group, and the corresponding treatment was given until the wound healed or 12 weeks had passed. At each time of debridement and dressing change, a digital camera was used to take pictures (at least once a week) to measure the area of foot ulcers; ABI measurement, pain assessment (Visual-Analog Scale scale) are performed at regular intervals. Follow-up visits were performed at the 4th, 8th, 12th, 24th, and 52nd weeks of treatment to evaluate wound healing, ulcer recurrence, and amputation.

（8） Outcome measures

Main outcome measures: 12-week wound healing rate

Secondary outcomes: 12-week ulcer area reduction rate, ulcer healing time, ulcer recurrence rate, amputation rate, pain assessment, and other adverse effects.

（9） Statistical analysis

Stata 13 was used for statistical analysis. The primary outcome measure was the chi-square test to assess the wound healing rate at 12 weeks. Logistc regression analysis was used to analyze possible confounding factors affecting wound healing. Secondary outcome measures: The 12-week ulcer area reduction rate and ulcer healing time were assessed by the independent samples t-test, and the ulcer recurrence rate and amputation rate were assessed by chi-square test. At the same time, Kaplan-Meire survival analysis was used to compare wound healing between the two groups. $P < 0.05$ was statistically significant.

4. Subject screening

1. Inclusion Criteria

(1) Be between the ages of 18 and 80; (2) From the plateau area (the place of residence is above 2500m above sea level); (3) Chronic refractory wounds (wounds that have not healed for 4 weeks): diabetic chronic lower limb skin ulcers, lower limb

venous insufficiency ulcers; (4) Appropriate blood supply, ABI $\geqslant$ 0.6, transcutaneous partial pressure of oxygen > 30mmHg; (5) Ulcer area 1-20cm2.

2. Exclusion Criteria

(1) gangrene of the limbs; (2) osteomyelitis; (3) malignant tumors; (4) HIV-positive; (5) Serious heart, liver, kidney, respiratory system, nervous system and other diseases; (6) Long-term use of steroids or other immunosuppressants; (7) Pregnant or women who plan to become pregnant 3 months before or after treatment, and who are breastfeeding; (8) Those who have mental illness or severe cognitive impairment, alcohol or drug abuse, and cannot cooperate with treatment.

3. Termination Study Criteria

(1) The patient voluntarily withdrew from the trial; (2) Those who have serious adverse reactions that lead to treatment interruption; (3) Those who did not respond to ulcer treatment after 4 weeks of treatment at the beginning of the trial (ulcer area reduction <20%); (4) The wound shows signs of serious clinical infection and requires immediate surgical intervention; (5) Those who have major toe amputation/limb indication in the affected limb where the target lesion is located.

5. Alternate treatment

In recent years, a variety of local treatment methods for chronic wounds, such as negative pressure suction closed drainage, recombinant human epidermal growth factor, murine nerve fibroblast growth factor, autologous platelet-rich gel, etc., have been gradually applied in clinical practice, but the treatment effect is still limited and the treatment cost is high.

6. Test items and test time

In this study, the wound area was measured regularly (1, 2, 4, 8, 12 weeks) to evaluate the efficacy.

7. Efficacy evaluation criteria

1. Effectiveness evaluation: according to the wound healing rate and healing time.

Wound area was measured and wound healing rate was calculated weekly before and after treatment. The wound area is directly calculated or digitally photographed using the sterile standard transparent checkered film, and the wound area of the digital photography is automatically calculated by the Image J medical image analysis software computer.

(1) Wound healing rate = (initial area of treatment - area after treatment) / initial area of treatment * 100%. Healing effect: (1) The healing rate of wounds is ≥90%; (2) 60% effective ≤ wound healing rate <90%; (3) 30% improvement≤ wound healing rate <60%; (4) Ineffective, wound healing rate<30%

(2) Healing time: the time when the wound is completely covered by the epithelium (days)

2. Safety evaluation:

(1) All adverse reactions were recorded.

(2) Incidence of adverse reactions = number of patients with an adverse reaction / total number of patients in the group * 100%

8. Observation, recording and management of adverse events

Local wound oxygen therapy has been used to treat acute and chronic wounds since the 60s of the 20th century. The technology for the design and manufacture of lower limb wound oxygen therapy devices has matured. Since this is a non-invasive treatment that provides slightly more pressure than the ambient pressure (0-50mbar), it is a pain-free, non-invasive, and safe treatment for patients.

1. Before treatment: Clean and disinfect the oxygen therapy cabin, foot cover or disposable limb oxygen therapy bag of the lower limb wound oxygen therapy device to reduce any potential risk of cross-infection. It is advisable to remove bandages, dressings or ointments attached to the wound before treatment. Wounds should be cleaned or debrided according to standard wound care methods prior to treatment with the device.

2. Treatment: Set the treatment time and treatment pressure according to the doctor's prescription: (1) If the treatment pressure exceeds the limit range, the

equipment will send out a high pressure alarm, the valve will be closed immediately, and the cabin pressure will be discharged into the atmosphere and automatically relieved. (2) If the treatment pressure in the cabin is not reached within 90 seconds, indicating that there is an air leak in the equipment, a low pressure alarm will be issued, and it is necessary to check whether all the pipes are connected correctly; Check the tightness of the boot and the tightness of the hatch and replace it if necessary; Adjust the patient's position appropriately. (3) Ensure that the room is ventilated during treatment, and do not smoke, moxibustion or use open flames. (4) During the treatment period, the patient should be assessed and debrided, and the wound photos should be collected, and the treatment situation should be recorded.

3. Post-treatment: Patients should follow the clinician's advice, use appropriate dressings according to standard nursing procedures, change dressings on time, and do a good job of follow-up feedback after treatment. Properly dispose of disposable foot covers or disposable limb oxygen therapy bags. The interior and exterior of the disinfection chamber and the external surfaces of the regulator, including the air and oxygen hoses and power cords, are cleaned and disinfected in accordance with the manufacturer's requirements.

If an adverse reaction or serious adverse reaction occurs, the adverse reaction will be reported and treated in accordance with the law.

9. Quality control and quality assurance of the study

The research team will strictly carry out the testing of laboratory indicators, strictly implement the implementation requirements of SOP and GCP regulations, conduct strict training for researchers, strive to improve subject compliance, collect data accurately, truthfully, completely and in a timely manner, reasonably organize and analyze data, monitor the research process in real time, and complete a summary report.

10. Data security monitoring

The clinical study will develop a data security monitoring plan according to the size of the risk. All adverse events are recorded in detail, properly handled and tracked until properly resolved or the condition is stable, and serious adverse events and unexpected events are reported to the ethics review committee, competent authorities, sponsors and drug regulatory departments in a timely manner in accordance with regulations; The principal investigator regularly conducted a cumulative review of all adverse events, and convened investigator meetings to evaluate the risks and benefits of the study if necessary; Double-blind trials can be urgently unblinded if necessary to ensure the safety and rights of subjects.

11. Statistical analysis

Stata 13 was used for statistical analysis. The primary outcome measure was the chi-square test to assess the wound healing rate at 12 weeks. Logistc regression analysis was used to analyze possible confounding factors affecting wound healing. Secondary outcome measures: The 12-week ulcer area reduction rate and ulcer healing time were assessed by the independent samples t-test, and the ulcer recurrence rate and amputation rate were assessed by chi-square test. At the same time, Kaplan-Meire survival analysis was used to compare wound healing between the two groups. $P < 0.05$ was statistically significant.

12. Ethical principles and requirements for clinical research

Clinical research will follow the relevant provisions of the Declaration of Helsinki of the World Medical Conference and the Measures for the Ethical Review of Biomedical Research Involving Humans of the National Health and Family Planning Commission of the People's Republic of China, and specifically implement the principles and requirements of informed consent, privacy protection, free and

compensation for research, risk control, protection of special subjects and compensation for research-related damages. The clinical study is not carried out until the study is approved by the Ethics Review Committee prior to the start of the study. Before each subject is enrolled in this study, the investigator has the responsibility to give the subject or/and his legal representative a complete and comprehensive introduction to the purpose, procedures and possible risks of this study, and sign a written informed consent form, and should let the subjects know that their participation in the clinical study is completely voluntary, they can refuse to participate or withdraw from the study at any stage of the trial at any time without discrimination and retaliation, and their medical treatment and rights and interests will not be affected. The informed consent form should be retained as a clinical research document for future reference, so as to effectively protect the personal privacy and data confidentiality of the subjects.

13. **Research progress**

2024.10- 2025.10: Patients with chronic refractory wounds who meet the research conditions for a long time will be screened and recruited to be included in the study after signing informed consent. According to the research plan, the baseline data of the subjects were comprehensively collected, including demographic data, comorbid diseases and complications, biochemical indicators and other examinations. According to the post-enrollment plan, corresponding intervention treatment was carried out for the patients, and the wound healing rate, healing time and other outcome indicators were evaluated.

2025.10-2026.04: Continue to recruit eligible subjects and improve the study. For the patients included in the study, the follow-up was carried out according to the preset follow-up time to evaluate the wound recurrence rate and amputation rate after intervention treatment.

2026.04-2026.07: Continue to improve the follow-up of cases. Analyze the experimental results, analyze the data, write summary materials and papers, and carry out the project conclusion and achievement appraisal.

14. **Participants**

name	job title	specialized	task	GCP Training Certificate
Ran Xingwu	Chief physician	incretion	Schematic design and technical guidance	Yes
David Chen	Deputy Chief Physician	incretion	Screening and treatment	Yes
Gao Yun	Deputy Chief Physician	incretion	Screening and treatment	Yes
Chen Lihong	Attending	incretion	Screening and treatment	Yes
Li Yan	Research Assistant	incretion	Screening and treatment	Yes
Wu Jing	not	incretion	Screening and follow-up	Yes
Fang Yixuan	not	incretion	Screening and follow-up	Yes

15、References

1 Liao Xincheng, Guo Guanghua. Classification and clinical evaluation of chronic refractory wounds. *Chinese Journal of Injury and Repair: Electronic Edition* 2017:303-5.

2 Cheng B, Jiang Y, Fu X, Hao D, Liu H, Liu Y, et al. Epidemiological characteristics and clinical analyses of chronic cutaneous wounds of inpatients in China: Prevention and control. *Wound Repair Regen* 2020;**28**:623-30.

3 He Si, Yan Chenyuan, Gang Qiaojian, Jia Caixia, Medical Review Ha J. Pathological changes and characteristics of refractory wounds in plateau areas. 2022;**28**:2746-50.

4 Qi Wanle, Zhuo Mojia, Tian Yan, Dawa Zhuoma, Ma Ziying, An Yanan, et al. Epidemiological investigation and analysis of elderly patients with chronic refractory wounds in the plateau area. *Chinese Journal of Injury and Repair: Electronic Edition* 2021;**16**:6.

5 Yu Xiaohui, Ding Sijuan, He Chong, Feng Dongfang, Xiao Jing, Chen Hong, et al. Analysis of the distribution characteristics and drug resistance of common culture bacteria in burn patients in the plateau hypoxic environment. 2022;**43**:2841-45.

6 Lian Guofeng, Chen Yu, Chen Xingshu, People's Military Doctor Luo J. A series of studies on the prevention and control of plateau diseases and disasters in the army(7) Geographical characteristics and prevention measures of poor wound healing in plateau areas. 2019:93-6.

7 Gottrup F, Dissemond J, Baines C, Frykberg R, Jensen P, Kot J, et al. Use of Oxygen Therapies in Wound Healing. *J Wound Care* 2017;**26**:S1-s43.

8 Chen L, Gao Y, Li Y, Wang C, Chen D, Gao Y, et al. Severe Intermittent Hypoxia Modulates the Macrophage Phenotype and Impairs Wound Healing Through Downregulation of HIF-2α. *Nature and*

science of sleep 2022;**14**:1511-20.
9 Kranke P, Bennett MH, Martyn-St James M, Schnabel A, Debus SE, Weibel S. Hyperbaric oxygen therapy for chronic wounds. *Cochrane Database Syst Rev* 2015;**2015**:Cd004123.
10 Gordillo GM, Roy S, Khanna S, Schlanger R, Khandelwal S, Phillips G, et al. Topical oxygen therapy induces vascular endothelial growth factor expression and improves closure of clinically presented chronic wounds. *Clin Exp Pharmacol Physiol* 2008;**35**:957-64.
11 Fries RB, Wallace WA, Roy S, Kuppusamy P, Bergdall V, Gordillo GM, et al. Dermal excisional wound healing in pigs following treatment with topically applied pure oxygen. *Moved Nothing* 2005;**579**:172-81.
12 Asmis R, Qiao M, Zhao Q. Low flow oxygenation of full-excisional skin wounds on diabetic mice improves wound healing by accelerating wound closure and reepithelialization. *Int Wound J* 2010;**7**:349-57.
13 Tawfick W, Sultan S. Does topical wound oxygen (TWO2) offer an improved outcome over conventional compression dressings (CCD) in the management of refractory venous ulcers (RVU)? A parallel observational comparative study. *Eur J Vasc Endovasc Surg* 2009;**38**:125-32.
14 Blackman E, Moore C, Hyatt J, Railton R, Frye C. Topical wound oxygen therapy in the treatment of severe diabetic foot ulcers: a prospective controlled study. *Ostomy Wound Manage* 2010;**56**:24-31.
15 Frykberg RG, Franks PJ, Edmonds M, Brantley JN, Téot L, Wild T, et al. A Multinational, Multicenter, Randomized, Double-Blinded, Placebo-Controlled Trial to Evaluate the Efficacy of Cyclical Topical Wound Oxygen (TWO2) Therapy in the Treatment of Chronic Diabetic Foot Ulcers: The TWO2 Study. *Diabetes Care* 2020;**43**:616-24.
