# Supplementary material for: The efficacy and safety of topical wound oxygen therapy for chronic refractory wounds at high altitude: Protocol for a randomized controlled clinical trial
Source: PLoS One. 2025 Jul 10;20(7):e0324475. doi: 10.1371/journal.pone.0324475 (PMC12244751; doi:10.1371/journal.pone.0324475)
Supplement: Attachment — Submitted filename: The efficacy and safety of TOT.pdf [file pone.0324475.s005.pdf]

The efficacy and safety of topical wound oxygen therapy for chronic refractory wounds at high altitude: protocol for a randomized controlled clinical trial
Study Protocol

Generally:

This study is well-structured and scientifically sound, incorporating an appropriate methodology to assess **TOT in high-altitude wound care**. To strengthen its impact, additional consideration of **alternative treatments, environmental confounders, accessibility concerns, and subgroup analyses** is recommended.

Abstract:

Strengths:

The title is clear and directly conveys the study's focus. The abstract concisely summarizes the rationale, methodology, and expected impact, making it accessible to readers.

Comments/Recommendations:

The abstract could briefly highlight potential limitations to set appropriate expectations for the study's findings.
Consider incorporating a sentence acknowledging challenges related to study execution, such as patient adherence or environmental variability.

Introduction:

Strengths:

The introduction effectively contextualizes the problem by discussing high-altitude physiology and chronic wound characteristics. The rationale for **Topical Wound Oxygen Therapy (TOT)** is well-articulated.

Comments/Recommendations:

The background lacks discussion on existing alternative treatments in high-altitude wound care, which could add depth. Expanding the introduction to compare TOT with other wound healing strategies in high-altitude settings could improve the justification for the study.

Methods

Strengths:

The study design is rigorous, following a **randomized, double-blinded, placebo-controlled trial** structure. The inclusion/exclusion criteria are clearly stated, ensuring patient selection aligns with study objectives. Ethical considerations are thorough, with adherence to international clinical trial guidelines. The sample size estimation methodology is clearly justified, incorporating expected dropout rates for a robust trial.

Comments/Recommendations:

- Wound follow up (assessment tool) validity and reliability???
- Limited information on how environmental factors (temperature, altitude fluctuations) could influence the results. Consider adding a section discussing how environmental variables will be monitored and controlled to ensure consistency in patient conditions.
- The dissemination plan focuses on peer-reviewed publications but does not mention stakeholder engagement for policy translation. Consider integrating details about how study findings will be communicated to healthcare providers or policymakers, potentially impacting wound care practices in plateau medicine.
- While statistical analysis methods are well-articulated, confounding variables such as **patient comorbidities or socioeconomic factors** affecting adherence to treatment are not explicitly addressed. Add a discussion about potential confounders and how the analysis will account for them, ensuring findings remain reliable.

Results (Pending Data Collection)

Strengths:

- The study is designed to collect both primary and secondary outcome measures, providing a comprehensive evaluation of wound healing efficacy.

Comments/Recommendations:

- No clear mention of a **subgroup analysis**, which could reveal variations in response to TOT based on age, severity of wounds, or underlying health conditions. Consider predefining patient subgroups to analyze differential effects of TOT, enhancing personalized treatment recommendations.

Discussion

Strengths:

- The discussion highlights the significance of TOT in chronic wound care, referencing existing literature and emphasizing the novelty of testing it in high-altitude populations.

Comments/Recommendations:

- The discussion does not sufficiently address **potential limitations of TOT**, such as accessibility of the therapy in resource-limited settings. Adding a section on potential **barriers to implementation** (e.g., cost, equipment availability, training requirements for healthcare professionals) would make the discussion more practical.

Conclusion

Strengths:

- The conclusion ties back to the study's objective and reinforces the potential clinical relevance of TOT.

Comments/Recommendations:

- Could benefit from a brief note on future research directions beyond high-altitude chronic wounds. Suggesting further studies on **TOT's mechanism of action or its applicability in different wound types** would enhance the impact of the conclusion.

Recommendations chapter not included at all:

- Provide clearer recommendations for institutional adoption of student-led career experience activities.
- Summarize practical ways for educators to use findings in curriculum development.

References:

Strengths:

- **Relevant Literature**: The references provide a solid foundation for the study, particularly in wound healing, hypoxia, and topical oxygen therapy.

- **Clinical Trial Support**: Several cited studies, such as **Frykberg et al. (2020)** and **Blackman et al. (2010)**, reinforce the therapeutic potential of **TOT**.

- **Diversity in Citations**: The mix of **Chinese and international studies** ensures that regional wound care challenges are adequately addressed while maintaining global relevance.

Comments/Recommendations:

- **Limited High-Altitude-Specific References**: Although the study is focused on plateau medicine, there are relatively few citations specifically exploring **chronic wound healing in high-altitude regions**.

- **Potential Gaps in Recent Literature**: Some references date back several years. Integrating the latest findings, particularly in **biotechnology and oxygen therapy innovations**, could enhance the study's scientific rigor.
- **Expand on High-Altitude Wound Research**: Incorporating **recent meta-analyses** or studies focused on **hypoxia-induced healing impairments** in plateau settings would strengthen the argument.
- **Include Policy-Oriented References**: If **clinical adoption and accessibility** are considerations, referencing reports on **oxygen therapy infrastructure in remote regions** would improve practical relevance.
- **Ensure Consistency in Citation Format**: While the references appear well-structured, a quick review for **uniformity in formatting** (e.g., DOI links, full journal names) would enhance clarity.
