# Supplementary material for: The efficacy and safety of topical wound oxygen therapy for chronic refractory wounds at high altitude: Protocol for a randomized controlled clinical trial
Source: PLoS One. 2025 Jul 10;20(7):e0324475. doi: 10.1371/journal.pone.0324475 (PMC12244751; doi:10.1371/journal.pone.0324475)
Supplement: Attachment — Submitted filename: PONE-D-25-21928 Allen review.docx [file pone.0324475.s006.docx]

In The efficacy and safety of topical wound oxygen therapy for chronic refractory wounds at high altitude: protocol for a randomized controlled clinical trial, Lin et al. describe a well thought out protocol for a clinical evaluation for TOT therapy. The authors do a good job explaining the rationale and need for the study. The experimental design, inclusion and exclusion criteria, and evaluation plan seem appropriate for the study. Overall, this protocol is appropriate for publication with minor revisions.

Background

- Briefly introduce the basic mechanism of TOT therapy

Methods

- Briefly describe the rationale behind therapy regimen. I.e. is the timing and O2 concentration based upon animal trials? Are there any considerations for implementing in human patients?
