# Supplementary material for: The efficacy and safety of topical wound oxygen therapy for chronic refractory wounds at high altitude: Protocol for a randomized controlled clinical trial
Source: PLoS One. 2025 Jul 10;20(7):e0324475. doi: 10.1371/journal.pone.0324475 (PMC12244751; doi:10.1371/journal.pone.0324475)
Supplement: Attachment — Submitted filename: Response to Reviewers.docx [file pone.0324475.s007.docx]

Dear editor,

We extend our gratitude to the Editor and Reviewers for their valuable time and constructive feedback. We highly appreciate the opportunity to resubmit our manuscript following the journal’s requirements. In response, we have thoroughly addressed all the comments raised by the Journal. Additionally, our detailed point-by-point replies to the Reviewers’ comments are provided below. Tracked changes are provided in the Revised Manuscript.

**Reviewer #1**: The efficacy and safety of topical wound oxygen therapy for chronic refractory wounds at high altitude: protocol for a randomized controlled clinical trial
Study Protocol

Generally:
This study is well-structured and scientifically sound, incorporating an appropriate methodology to assess TOT in high-altitude wound care. To strengthen its impact, additional consideration of alternative treatments, environmental confounders, accessibility concerns, and subgroup analyses is recommended.
1. **Abstract**:
Strengths:
The title is clear and directly conveys the study’s focus. The abstract concisely summarizes the rationale, methodology, and expected impact, making it accessible to readers.
Comments/Recommendations:
The abstract could briefly highlight potential limitations to set appropriate expectations for the study's findings.
Consider incorporating a sentence acknowledging challenges related to study execution, such as patient adherence or environmental variability.

**Response**: Thank you for your feedback. We made revisions to discuss the limitations and challenges of this study. However, we placed these descriptions in the discussion section.

*“it should be emphasized that the implementation of TOT therapy requires the availability of TOT device, which may pose challenges in resource-limited settings. In fact, the procedure for TOT therapy is relatively straightforward and can be effectively performed by healthcare practitioners following minimal training.”*

*“The single-center design of this study may impose limitations on its external validity. Further multi-center studies could potentially strengthen the generalizability and reliability of these findings”*

*“Of course, it should be noted that it may be a challenge to recruit enough subjects due to the lower population density at high altitudes.”*

2. **Introduction**:

Strengths:

The introduction effectively contextualizes the problem by discussing high-altitude physiology and chronic wound characteristics. The rationale for Topical Wound Oxygen Therapy (TOT) is well-articulated.

Comments/Recommendations:

The background lacks discussion on existing alternative treatments in high-altitude wound care, which could add depth. Expanding the introduction to compare TOT with other wound healing strategies in high-altitude settings could improve the justification for the study.

**Response**: Thank you for your feedback. We have incorporated an alternative treatment for chronic wounds in the Background section.

*“However, the recommendations for caring for chronic wounds at high altitudes are not substantially distinct from those in plains. Limited literature has been published to investigate interventions that could enhance wound healing in high-altitude environments.”*

*“Standard of care for chronic wounds includes off-loading, anti-infection strategies, debridement, local wound care, negative-pressure wound therapy, and the application of various dressings. Interventions tailored to address the pathophysiology of wounds at high altitudes may offer promising avenues for improving wound healing outcomes.”*

3. **Methods**

Strengths:

The study design is rigorous, following a randomized, double-blinded, placebo-controlled trial structure. The inclusion/exclusion criteria are clearly stated, ensuring patient selection aligns with study objectives. Ethical considerations are thorough, with adherence to international clinical trial guidelines. The sample size estimation methodology is clearly justified, incorporating expected dropout rates for a robust trial.

Comments/Recommendations:

• Wound follow up (assessment tool) validity and reliability???

• Limited information on how environmental factors (temperature, altitude fluctuations) could

influence the results. Consider adding a section discussing how environmental variables will be monitored and controlled to ensure consistency in patient conditions.

• The dissemination plan focuses on peer-reviewed publications but does not mention stakeholder engagement for policy translation. Consider integrating details about how study findings will be communicated to healthcare providers or policymakers, potentially impacting wound care practices in plateau medicine.

• While statistical analysis methods are well-articulated, confounding variables such as patient comorbidities or socioeconomic factors affecting adherence to treatment are not explicitly addressed. Add a discussion about potential confounders and how the analysis will account for them, ensuring findings remain reliable.

**Response**: Thank you. During the follow-up period, we will use objective imaging techniques to document the wound condition. Consequently, the follow-up assessment of the wound is expected to exhibit high validity and reliability. To maintain consistency in patient conditions, we will exclusively recruit individuals residing in areas with an elevation of at least 2500 meters above sea level. In addition, we will plan to disseminate the findings of this study to healthcare providers and policymakers through presentations at academic conferences. Additionally, the manuscript has been revised to clearly outline the covariables that require adjustment.

*“Digital photography will be employed to systematically document images on a weekly basis. The Image J software will be utilized to quantitatively measure the area of the foot ulcer during each debridement and dressing change procedure”*

*“Participants are required to reside in regions with an elevation of no less than 2,500 meters above sea level”*

*“The findings from this study will be disseminated through publication in relevant, peer-reviewed scientific journals by the research team. In addition, the research team will actively communicate the findings of the study with healthcare providers and policymakers at academic conferences.”*

*“Confounding factors, such as sex, age, history of diabetes, ulcer categories, wound area, and infection status, will be adjusted to ensure the reliability of the findings.”*

4. **Results** (Pending Data Collection)

Strengths:

• The study is designed to collect both primary and secondary outcome measures, providing a

comprehensive evaluation of wound healing efficacy.

Comments/Recommendations:

• No clear mention of a subgroup analysis, which could reveal variations in response to TOT based on age, severity of wounds, or underlying health conditions. Consider predefining patient subgroups to analyze differential effects of TOT, enhancing personalized treatment recommendations.

**Response**: Thank you.

*“Subgroup analysis will be conducted according to age, wound categories, and wound area.”*

5. **Discussion**

Strengths:

• The discussion highlights the significance of TOT in chronic wound care, referencing existing literature and emphasizing the novelty of testing it in high-altitude populations.

Comments/Recommendations:

• The discussion does not sufficiently address potential limitations of TOT, such as accessibility of the therapy in resource-limited settings. Adding a section on potential barriers to implementation (e.g., cost, equipment availability, training requirements for healthcare professionals) would make the discussion more practical.

**Response**: Thank you. We have made revisions accordingly.

*“However, it is important to highlight that the implementation of TOT therapy requires the availability of TOT device, which may pose challenges in resource-limited settings. In fact, the procedure for TOT therapy is relatively straightforward and can be effectively performed by healthcare practitioners following minimal training.”*

6. **Conclusion**

Strengths:

• The conclusion ties back to the study’s objective and reinforces the potential clinical relevance of TOT.

Comments/Recommendations:

• Could benefit from a brief note on future research directions beyond high-altitude chronic wounds.

Suggesting further studies on TOT’s mechanism of action or its applicability in different wound types would enhance the impact of the conclusion.

Recommendations chapter not included at all:

• Provide clearer recommendations for institutional adoption of student-led career experience

activities.

• Summarize practical ways for educators to use findings in curriculum development.

**Response**: Thank you.

*“Further studies could be conducted to clarity the mechanism of TOT therapy and its applicability across various wound categories.”*

7. **References**:

Strengths:

• Relevant Literature: The references provide a solid foundation for the study, particularly in wound healing, hypoxia, and topical oxygen therapy.

• Clinical Trial Support: Several cited studies, such as Frykberg et al. (2020) and Blackman et al. (2010), reinforce the therapeutic potential of TOT. • Diversity in Citations: The mix of Chinese and international studies ensures that regional wound care challenges are adequately addressed while maintaining global relevance.

Comments/Recommendations:

• Limited High-Altitude-Specific References: Although the study is focused on plateau medicine, there are relatively few citations specifically exploring chronic wound healing in high-altitude regions.

• Potential Gaps in Recent Literature: Some references date back several years. Integrating the latest findings, particularly in biotechnology and oxygen therapy innovations, could enhance the study’s scientific rigor.

• Expand on High-Altitude Wound Research: Incorporating recent meta-analyses or studies focused on hypoxia-induced healing impairments in plateau settings would strengthen the argument.

• Include Policy-Oriented References: If clinical adoption and accessibility are considerations, referencing reports on oxygen therapy infrastructure in remote regions would improve practical relevance.

• Ensure Consistency in Citation Format: While the references appear well-structured, a quick

review for uniformity in formatting (e.g., DOI links, full journal names) would enhance clarity.

**Response**: Thank you for your feedback. We have revised the references in the manuscript according to the provided comments. Specifically, five additional references have been incorporated, and several outdated citations have been replaced with more recent studies to ensure the content reflects the latest developments in the field.

**Reviewer #2**: This study addresses an important and underexplored clinical problem — the management of chronic refractory wounds in high-altitude populations using topical wound oxygen therapy (TOT). The proposed randomized, double-blinded, placebo-controlled design is methodologically robust, and the outcomes chosen are relevant and clinically significant. However, several key areas require revision to improve the protocol's scientific rigor, reproducibility, and applicability:

1. Wound Type Stratification:
The inclusion of multiple chronic wound types (e.g., diabetic foot ulcers, venous ulcers) without subgroup stratification or analysis is a significant limitation. Different wound etiologies have distinct pathophysiology and may respond differently to oxygen therapy. The authors should consider stratified randomization or include planned subgroup analyses.

**Response**: Thank you. We will conduct a subgroup analysis according to the different wound types.

*“Confounding factors, such as sex, age, history of diabetes, ulcer categories, wound area, and infection status, will be adjusted to ensure the reliability of the findings. Subgroup analysis will be conducted according to age, wound categories, and wound area.”*

2. Standard of Care Consistency:
"Standard wound care" is left to physician discretion without a defined protocol. This introduces variability and may affect the internal validity of the study. A minimum standard wound care protocol should be outlined (e.g., dressing selection, debridement practices, offloading strategies) to ensure consistency across groups.

**Response**: Thank you. We delineate the specific interventions that should be included in the “standard of care”. However, we did not explicitly detail the concrete procedures of these interventions.

*“all patients will receive standard medical care for chronic wounds, which includes the management of blood sugar, blood pressure, and lipid profiles; antiplatelet aggregation and anticoagulation therapies; infection prevention measures; promotion of physical activity; pressure relief interventions; and debridement procedures.”*

3. Blinding of Sham Therapy:
The manuscript lacks detail on how sham oxygen therapy (ambient air) will be made indistinguishable from the active TOT, which could compromise blinding. The authors should clarify how the control setup will simulate the sensory experience of TOT (e.g., pressure, temperature, sound).

**Response**: We revised the manuscript to explicitly state that ambient air will be used as the gas source in the sham therapy, while the procedure remains identical to the active TOT therapy.

*“Patients in the control group received standard wound care and sham therapy. The sham devices looked and operated identically with the active TOT therapy, with the sole difference being the use of ambient air as the gas source instead of an oxygen supply.”*

4. Patient-Centered Outcomes Missing:
Pain and quality of life are briefly mentioned but not evaluated using standardized tools. These outcomes are crucial in chronic wound studies. The authors are encouraged to incorporate validated instruments such as the Visual Analog Scale (VAS) for pain and the EQ-5D or Wound-QoL for quality of life.

**Response**: Thank you. We are appreciative of the opportunity to include the VAS (Visual Analog Scale) for pain and Wound-QoL as the secondary outcomes.

*“****Secondary outcome***

*1. Rate of ulcer area reduction: percent change in wound size after 12 weeks*

*2. Time to ulcer healing: duration until complete wound closure*

*3. One-year ulcer recurrence rate*

*4. Amputation rate at 12 weeks*

*5. VAS score: Visual analogue scale for pain assessment*

*6. Wound-QoL: Questionnaire assessing quality of life in patients with chronic wounds”*

5. Literature Review Imbalance:
The background heavily emphasizes positive studies supporting TOT, with little discussion of conflicting or negative findings. A more balanced review of current literature is necessary to frame the rationale objectively.

**Response**: Thank you for your feedback. We have reviewed the relevant literature and incorporated a detailed description of conflicting findings.
*“Specifically, while some studies suggest the efficacy of TOT therapy in wound healing, other studies report inconsistent results. Notably, the studies by Driver et al. and He et al. did not demonstrate any additional benefits of TOT therapy for wound healing compared to moist wound therapy.”*

6. Generalizability and Limitations:
While the setting is appropriate for the study goals, the single-center design limits broader applicability. This should be acknowledged explicitly in the discussion, with a recommendation for future multi-center research.

**Response**: Thank you for your feedback. In the discussion section, we have revised the text to state.

*“The single-center design of this study may impose limitations on its external validity. Further multi-center studies could potentially strengthen the generalizability and reliability of these findings.”*

7. Microbiological Context:
Given the noted differences in microbial profiles in high-altitude wounds, the protocol could be strengthened by including infection tracking or microbial analysis as exploratory endpoints.

**Response**: Thank you for your valuable suggestion. Taking microbial analysis into consideration is indeed of great significance. In our future analyses, we will endeavor to explore the impact of TOT therapy on microbial profiles in a more systematic manner.

8. Figures and Visuals:
Figures 1 and 2 are informative but visually dense. Consider simplifying and clarifying their design for better comprehension.
In conclusion, this is a promising and relevant study. With moderate revisions addressing the points above, it can make a valuable contribution to wound care research in resource-limited, high-altitude environments.

**Response**: Thank you for your feedback. We have made the revisions in accordance with the provided suggestions.

**Reviewer #3**: In The efficacy and safety of topical wound oxygen therapy for chronic refractory wounds at high altitude: protocol for a randomized controlled clinical trial, Lin et al. describe a well thought out protocol for a clinical evaluation for TOT therapy. The authors do a good job explaining the rationale and need for the study. The experimental design, inclusion and exclusion criteria, and evaluation plan seem appropriate for the study. Overall, this protocol is appropriate for publication with minor revisions.

Background
- Briefly introduce the basic mechanism of TOT therapy

**Response**: Thank you for your feedback. We have included a detailed description of the mechanism underlying TOT therapy.

*“Preclinical animal studies have shown that this therapy can increase transcutaneous oxygen partial pressure, upregulate vascular endothelial growth factor expression, enhance angiogenesis and tissue remodeling, and expedite wound healing”*

Methods
- Briefly describe the rationale behind therapy regimen. I.e. is the timing and O2 concentration based upon animal trials? Are there any considerations for implementing in human patients?

**Response**: Thank you. TOT therapy has previously been utilized in various clinical trials. In the present study, we have modified the interventional protocol accordingly.

*“The treatment involves the delivery of pressurized oxygen, with a pressure range of 0 to 50 mbar, generated by an oxygen concentrator operating at a flow rate of 10 L/min. Each session will last 90 minutes and will be conducted once daily, five days per week.”*

Reviewer #4: The primary objective of this randomized, double-blind, placebo-controlled clinical trial involving 250 residents living at high altitudes is to assess the efficacy and safety of topical wound oxygen therapy (TOT) for treating chronic refractory wounds. Participants will be randomly assigned to receive either TOT or sham oxygen therapy over a 12-week period, followed by a one-year follow-up. The primary endpoint is the wound healing rate at the end of the 12-week intervention. Secondary outcomes include reduction in ulcer area, time to complete healing, ulcer recurrence rate, amputation rate, pain levels, and the incidence of adverse events. If proven effective, TOT could serve as a valuable and safe adjunctive treatment for managing chronic wounds in high-altitude settings. The protocol covers all key aspects of study design, randomization, and statistical issues; however, there are several major statistical concerns about the current manuscript.

Statistical critiques:

1. Although the authors discuss power analysis and sample size estimation, they should explicitly clarify the following: (1) the statistical test used to determine the required sample size, (2) whether a one-sided or two-sided test was applied, and (3) that the reported 30% improvement refers to a relative, not absolute, increase in the wound healing rate. Specifically, the authors should state: “Assuming a baseline wound healing rate of 60% in the standard treatment group over a 12-week period, it is hypothesized that the addition of TOT will result in a 30% relative improvement in healing, i.e., 60% vs. 78%.”

**Response**: Thank you for your feedback. We have made the revisions as suggested.

*“the chi-square test will be utilized to evaluate the primary outcome measure, namely the wound healing rate at 12 weeks.”*

*“Assuming a baseline wound healing rate of 60% in the standard treatment group over a 12-week period, it is hypothesized that the incorporation of TOT would result in a 30% relative improvement in the wound healing rate, i.e., 60% vs. 78%. This hypothesis will be evaluated using a two-sided test with a significance level (α) of 0.05 and a statistical power (1-β) of 0.8.”*

2. Although the manuscript discusses randomization, the authors should explicitly specify the method used—for example, simple randomization (not recommended) or stratified permuted block randomization (highly recommended). If stratified permuted block randomization is chosen, the authors should clearly detail the stratification factor(s), block size, and other relevant parameters in the manuscript.

**Response**: Thank you for your feedback. We have made the revisions accordingly.

*“The random sequence will be generated by a researcher utilizing R software with the simple randomization method.”*

3. The statistical analysis plan covers the major outcomes but lacks essential methodological clarity and precision. While using the chi-square test for comparing wound healing rates is reasonable, the authors should clarify whether assumptions will be assessed and whether Fisher’s exact test will be used for sparse data. The proposed use of logistic regression to adjust for confounders is appropriate, but the model’s covariates, selection criteria, and diagnostics are not specified. Additionally, time-to-healing is better analyzed with survival methods rather than the proposed t-test, and the mention of Kaplan-Meier analysis is vague—for example, there is no indication of what time-to-event outcome is being evaluated or whether log-rank tests or Cox models will be used.

**Response**: Thank you for your feedback. We have made the revisions accordingly.

*“Specifically, the chi-square test will be employed to evaluate the primary outcome measure, namely the wound healing rate at 12 weeks. For sparse data, Fisher’s exact test will be applied.” “Logistic regression analysis will be conducted to investigate potential confounding factors that could influence wound healing. Confounding factors, such as sex, age, history of diabetes, ulcer categories of ulcer, wound area, and infection status, will be adjusted to ensure the reliability of the findings.”*

*“Kaplan-Meire survival analysis will be performed to compare wound healing between the two groups, with the log-rank test used for statistical comparison. Unadjusted and adjusted Cox proportional hazards models will be employed to evaluate the association between TOT therapy and wound healing. Multivariable models were adjusted for covariates such as age, sex, history of diabetes, ulcer categories, wound area, and infection status.”*

4. In summary, several important elements are missing from the current statistical analysis plan. There is no mention of how missing data will be addressed (a formal multiple imputation approach is recommended), whether analyses will follow the intention-to-treat principle, or how multiple testing across secondary endpoints will be controlled (either Bonferroni adjustment or false discovery rate [FDR] should be applied). The t-tests for ulcer area and healing time assume no severe skewness (skewness < 1), which should be assessed; otherwise, non-parametric methods such as the Wilcoxon rank-sum test should be used. To improve the rigor of the plan, the authors should clarify test assumptions, specify covariates, define outcomes more precisely, and incorporate appropriate strategies for handling missing data and multiplicity.

**Response**: Thank you for your feedback. We have made the revisions accordingly.

*“Multiple imputation approach will be utilized to address missing data.”*

*“Statistical analysis will be conducted using an intention-to-treat approach.”*

*“The reduction rate of ulcer area and wound healing time at 12 weeks were evaluated using either the independent t-test or the Mann-Whitney U test, contingent upon meeting the assumptions for parametric analysis.”*
