# Supplementary material for: The efficacy and safety of topical wound oxygen therapy for chronic refractory wounds at high altitude: Protocol for a randomized controlled clinical trial
Source: PLoS One. 2025 Jul 10;20(7):e0324475. doi: 10.1371/journal.pone.0324475 (PMC12244751; doi:10.1371/journal.pone.0324475)
Supplement: Attachment — Submitted filename: Response_to_Reviewers_auresp_2.docx [file pone.0324475.s008.docx]

Dear editor,

We thank the Editor and Reviewers for their valuable time and constructive feedback. We have reviewed the reference list and we are sure that there were no papers that have been retracted. And we also make some revisions to ensure it is complete and correct. The detailed point-by-point replies to the Reviewers’ comments are provided below.

Reviewer #2: 
Minor suggestions include:

Enhancing standardization in how “standard wound care” will be monitored or audited across patients.

Clarifying if outcome adjudicators for wound healing will be blinded or centralized.

Confirming the data repository platform intended for post-trial public data availability.

**Response**: Thank you for your suggestion. We have made corresponding revisions. Because the outcomes of wound healing were objective, the outcome adjudicators for wound healing will not be blinded or centralized. The data collected in the study will be stored in the hospital. The dataset will be available from the corresponding author on reasonable request.

*“To ensure that all patients received standard wound care, a checklist with the abovementioned contents will be used in the study.”*

*“The researchers will determine whether the outcomes were reached. Because of the objective feature, the outcome assessment for wound healing will not be blinded or centralized.”*

*“The data collected in the study will be stored in the hospital.* *The primary investigator has the access to the final dataset.* *The dataset without identifiable information will be available from the corresponding author on reasonable request.”*

Reviewer #4: The authors have responded well to the statistical issues raised in the previous review. There is no further statistical concern about this revised manuscript.

**Response**: Thank you.
